# Supplementary material for: Enhancing the prediction of vitamin D deficiency levels using an integrated approach of deep learning and evolutionary computing
Source: PeerJ Comput Sci. 2025 Feb 21;11:e2698. doi: 10.7717/peerj-cs.2698 (PMC11888904; doi:10.7717/peerj-cs.2698)
Supplement: Supplemental Information 3 [file peerj-cs-11-2698-s003.pdf]

Vitamin D Deficiency Risk Assessment Questionnaire

Participant Information

- **Patient ID:** ____________________
- **Age (years):** ____________________
- **Sex:**
  - ☐ Male
  - ☐ Female

Demographic Information

1. **Race/Ethnicity:**
   - ☐ African American
   - ☐ Asian
   - ☐ Hispanic
   - ☐ Caucasian
   - ☐ Other: ____________________
2. **Residential Area:**
   - ☐ Urban
   - ☐ Suburban
   - ☐ Rural
3. **Current Season (When completing this survey):**
   - ☐ Spring
   - ☐ Summer
   - ☐ Fall
   - ☐ Winter

Health and Lifestyle Information

4. **Body Mass Index (BMI):**
Height (in cm): ____________________
Weight (in kg): ____________________
Calculated BMI: ____________________

5. **Do you have any of the following medical conditions?**
   - ☐ Osteoporosis
   - ☐ Diabetes
   - ☐ Hypertension

- ☐ Cardiovascular disease
- ☐ None
- ☐ Other: ____________________

6. **Are you currently taking any medications? (Select all that apply)**
   - ☐ Anticonvulsants
   - ☐ Steroids
   - ☐ Diuretics
   - ☐ Vitamin D supplements
   - ☐ None
   - ☐ Other: ____________________
7. **Lifestyle Choices:**
   - Do you smoke?
     - ☐ Yes
     - ☐ No
   - Do you consume alcohol?
     - ☐ Yes
     - ☐ No
   - Do you engage in regular physical activity?
     - ☐ Yes
     - ☐ No

Dietary Intake
8. **Average daily intake of calcium (in mg):**
Please estimate your average calcium intake based on dairy products, supplements, and other sources.

- ☐ Less than 500 mg
- ☐ 500-1000 mg
- ☐ 1000-1500 mg
- ☐ More than 1500 mg

9. **Average daily intake of Vitamin D (in IU):**
   Please estimate your daily Vitamin D intake from food, supplements, and fortified products.
   - ☐ Less than 200 IU
   - ☐ 200-400 IU
   - ☐ 400-800 IU
   - ☐ More than 800 IU

Sun Exposure
10. **Average weekly sun exposure:**
How many hours per week do you spend outdoors with direct sun exposure?
- [ ] Less than 1 hour
- [ ] 1-3 hours
- [ ] 3-6 hours
- [ ] More than 6 hours

11. **In which parts of the day do you usually get sun exposure?**
    - ☐ Morning (before 10 AM)
    - ☐ Midday (10 AM to 3 PM)
    - ☐ Afternoon (after 3 PM)
12. **Do you use sunscreen regularly when outdoors?**
    - ☐ Yes
    - ☐ No

Vitamin D Levels and Diagnosis
13. **Most recent Vitamin D test result (if available):**
- *Vitamin D level (in ng/mL):* ____________________
- **Deficiency Level (if diagnosed by a physician):**
- [ ] Deficient
- [ ] Insufficient
- [ ] Sufficient
- [ ] Unsure

General Health Information
14. **In the past year, how often have you experienced fatigue or muscle pain?**
- [ ] Rarely
- [ ] Occasionally
- [ ] Frequently
- [ ] Constantly

15. **Do you have a history of bone fractures or low bone density?**
    - ☐ Yes
    - ☐ No

Additional Information
16. **Any other relevant health information or conditions not mentioned above?**
Please specify: ____

This detailed questionnaire gathers comprehensive information about a participant's demographics, lifestyle, health, and dietary habits, providing a strong foundation for the prediction of Vitamin D deficiency. Let me know if you'd like any adjustments or additional sections!
